# Supplementary material for: Unveiling the Bioactive Potential of the Invasive Jellyfish Phyllorhiza punctata Through Integrative Transcriptomic and Proteomic Analyses
Source: Biomolecules. 2025 Aug 4;15(8):1121. doi: 10.3390/biom15081121 (PMC12383608; doi:10.3390/biom15081121)
Supplement: Supplementary file 1 [file biomolecules-15-01121-s001.zip › Suplementary Material/FileS3/PEPFOLD4/XP_065059713_7812/clusters.html]

 PEP-FOLD clusters


Download archive of all models  here   

|  |  |  |
| --- | --- | --- |
| # | file | sOPEP |
| # cluster 1 - Size: 14 |  |  |
|  | XP\_065059713\_7812-model1 | -22.514 |
|  | XP\_065059713\_7812-model1.1 | -22.4247 |
|  | XP\_065059713\_7812-model1.2 | -22.4157 |
|  | XP\_065059713\_7812-model1.3 | -22.3949 |
|  | XP\_065059713\_7812-model1.4 | -22.3715 |
|  | XP\_065059713\_7812-model1.5 | -22.3343 |
|  | XP\_065059713\_7812-model1.6 | -22.321 |
|  | XP\_065059713\_7812-model1.7 | -22.3008 |
|  | XP\_065059713\_7812-model1.8 | -22.2905 |
|  | XP\_065059713\_7812-model1.9 | -22.2769 |
|  | XP\_065059713\_7812-model1.10 | -22.2326 |
|  | XP\_065059713\_7812-model1.11 | -22.1865 |
|  | XP\_065059713\_7812-model1.12 | -22.1865 |
|  | XP\_065059713\_7812-model1.13 | -22.1538 |
| # cluster 2 - Size: 23 |  |  |
|  | XP\_065059713\_7812-model2 | -22.4979 |
|  | XP\_065059713\_7812-model2.1 | -22.4747 |
|  | XP\_065059713\_7812-model2.2 | -22.3899 |
|  | XP\_065059713\_7812-model2.3 | -22.3899 |
|  | XP\_065059713\_7812-model2.4 | -22.3628 |
|  | XP\_065059713\_7812-model2.5 | -22.3067 |
|  | XP\_065059713\_7812-model2.6 | -22.2785 |
|  | XP\_065059713\_7812-model2.7 | -22.274 |
|  | XP\_065059713\_7812-model2.8 | -22.2619 |
|  | XP\_065059713\_7812-model2.9 | -22.2565 |
|  | XP\_065059713\_7812-model2.10 | -22.2565 |
|  | XP\_065059713\_7812-model2.11 | -22.2517 |
|  | XP\_065059713\_7812-model2.12 | -22.2435 |
|  | XP\_065059713\_7812-model2.13 | -22.2236 |
|  | XP\_065059713\_7812-model2.14 | -22.2225 |
|  | XP\_065059713\_7812-model2.15 | -22.1908 |
|  | XP\_065059713\_7812-model2.16 | -22.1818 |
|  | XP\_065059713\_7812-model2.17 | -22.1741 |
|  | XP\_065059713\_7812-model2.18 | -22.1612 |
|  | XP\_065059713\_7812-model2.19 | -22.1612 |
|  | XP\_065059713\_7812-model2.20 | -22.1196 |
|  | XP\_065059713\_7812-model2.21 | -22.1069 |
|  | XP\_065059713\_7812-model2.22 | -22.0123 |
| # cluster 3 - Size: 24 |  |  |
|  | XP\_065059713\_7812-model3 | -22.4751 |
|  | XP\_065059713\_7812-model3.1 | -22.4406 |
|  | XP\_065059713\_7812-model3.2 | -22.4136 |
|  | XP\_065059713\_7812-model3.3 | -22.4096 |
|  | XP\_065059713\_7812-model3.4 | -22.3829 |
|  | XP\_065059713\_7812-model3.5 | -22.3627 |
|  | XP\_065059713\_7812-model3.6 | -22.3326 |
|  | XP\_065059713\_7812-model3.7 | -22.3267 |
|  | XP\_065059713\_7812-model3.8 | -22.3133 |
|  | XP\_065059713\_7812-model3.9 | -22.3013 |
|  | XP\_065059713\_7812-model3.10 | -22.3004 |
|  | XP\_065059713\_7812-model3.11 | -22.2959 |
|  | XP\_065059713\_7812-model3.12 | -22.2917 |
|  | XP\_065059713\_7812-model3.13 | -22.289 |
|  | XP\_065059713\_7812-model3.14 | -22.259 |
|  | XP\_065059713\_7812-model3.15 | -22.2511 |
|  | XP\_065059713\_7812-model3.16 | -22.2462 |
|  | XP\_065059713\_7812-model3.17 | -22.2383 |
|  | XP\_065059713\_7812-model3.18 | -22.2087 |
|  | XP\_065059713\_7812-model3.19 | -22.1779 |
|  | XP\_065059713\_7812-model3.20 | -22.163 |
|  | XP\_065059713\_7812-model3.21 | -22.1544 |
|  | XP\_065059713\_7812-model3.22 | -22.1297 |
|  | XP\_065059713\_7812-model3.23 | -22.0489 |
| # cluster 4 - Size: 10 |  |  |
|  | XP\_065059713\_7812-model4 | -22.465 |
|  | XP\_065059713\_7812-model4.1 | -22.3851 |
|  | XP\_065059713\_7812-model4.2 | -22.3793 |
|  | XP\_065059713\_7812-model4.3 | -22.379 |
|  | XP\_065059713\_7812-model4.4 | -22.3203 |
|  | XP\_065059713\_7812-model4.5 | -22.3191 |
|  | XP\_065059713\_7812-model4.6 | -22.285 |
|  | XP\_065059713\_7812-model4.7 | -22.248 |
|  | XP\_065059713\_7812-model4.8 | -22.2316 |
|  | XP\_065059713\_7812-model4.9 | -22.0927 |
| # cluster 5 - Size: 12 |  |  |
|  | XP\_065059713\_7812-model5 | -22.4188 |
|  | XP\_065059713\_7812-model5.1 | -22.407 |
|  | XP\_065059713\_7812-model5.2 | -22.3653 |
|  | XP\_065059713\_7812-model5.3 | -22.3433 |
|  | XP\_065059713\_7812-model5.4 | -22.3322 |
|  | XP\_065059713\_7812-model5.5 | -22.32 |
|  | XP\_065059713\_7812-model5.6 | -22.2983 |
|  | XP\_065059713\_7812-model5.7 | -22.239 |
|  | XP\_065059713\_7812-model5.8 | -22.2257 |
|  | XP\_065059713\_7812-model5.9 | -22.2048 |
|  | XP\_065059713\_7812-model5.10 | -22.1652 |
|  | XP\_065059713\_7812-model5.11 | -22.1351 |
| # cluster 6 - Size: 7 |  |  |
|  | XP\_065059713\_7812-model6 | -22.4045 |
|  | XP\_065059713\_7812-model6.1 | -22.3999 |
|  | XP\_065059713\_7812-model6.2 | -22.3361 |
|  | XP\_065059713\_7812-model6.3 | -22.3361 |
|  | XP\_065059713\_7812-model6.4 | -22.2715 |
|  | XP\_065059713\_7812-model6.5 | -22.2502 |
|  | XP\_065059713\_7812-model6.6 | -22.178 |
| # cluster 7 - Size: 9 |  |  |
|  | XP\_065059713\_7812-model7 | -22.3944 |
|  | XP\_065059713\_7812-model7.1 | -22.336 |
|  | XP\_065059713\_7812-model7.2 | -22.3225 |
|  | XP\_065059713\_7812-model7.3 | -22.3151 |
|  | XP\_065059713\_7812-model7.4 | -22.2876 |
|  | XP\_065059713\_7812-model7.5 | -22.284 |
|  | XP\_065059713\_7812-model7.6 | -22.2677 |
|  | XP\_065059713\_7812-model7.7 | -22.2677 |
|  | XP\_065059713\_7812-model7.8 | -22.1923 |
| # cluster 8 - Size: 1 |  |  |
|  | XP\_065059713\_7812-model8 | -22.104 |
|  |  |  |
